# Two new species from two genera of the family Ampeliscidae Krøyer, 1842 (Crustacea, Amphipoda) from the Clarion-Clipperton Zone, Pacific Ocean

**DOI:** 10.3897/zookeys.1274.141891

**Published:** 2026-03-24

**Authors:** Rachael A. Peart, Eva C. D. Stewart

**Affiliations:** 1 New Zealand Institute of Earth Sciences Ltd (Earth Sciences New Zealand), Wellington, New Zealand New Zealand Institute of Earth Sciences Ltd (Earth Sciences New Zealand) Wellington New Zealand; 2 School of Ocean and Earth Sciences, University of Southampton, SO14 3ZH, Southampton, UK Natural History Museum London United Kingdom; 3 Life Sciences Department, Natural History Museum, London, Cromwell Road, SW7 5BD, South Kensington, UK University of Southampton Southampton United Kingdom

**Keywords:** Abyss, *
Byblis
*, *
Byblisoides
*, deep sea, Pacific Ocean

## Abstract

Ampeliscidae is a species-diverse family that inhabits oceans around the world at depths from the intertidal to the hadal zone of 6500 m. During a series of collection expeditions to the Clarion-Clipperton Zone, a number of specimens of the family Ampeliscidae were sampled from depths ranging from 4097 m to 4275 m. Included in these samples were two species unknown to science, *Byblis hortonae***sp. nov**. and *Byblisoides jazdzewskae***sp. nov**. Molecular and morphological examination provides descriptions of these species and keys to the deep water *Byblis* species and the world *Byblisoides* species.

## Introduction

The family Ampeliscidae Krøyer, 1842 is characterised by having a large robust body, generally strongly laterally compressed. A long dominant head with often two pairs of eyes (or one pair or eyes absent), and a pereopod 7 differing in form and size to pereopods 5 and 6, and a fused urosomite 2 and 3. Currently, there are four genera in the family Ampeliscidae: *Ampelisca* Krøyer, 1842; *Byblis* Boeck, 1871; *Byblisoides* K.H. Barnard, 1931 and *Haploops* Liljeborg, 1856, which are recorded from all the oceans of the world, especially the Pacific Ocean. The type genus *Ampelisca* is by far the most species-rich, with 210 species. The remaining three genera are considerably less diverse with *Byblis* having 79 species, *Byblisoides* with nine species, and *Haploops* with 30 species (Horton et al. 2024).

The depth range of different species can often be aligned with the genus. *Byblis*, *Byblisoides*, and *Haploops* have a mainly deeper and often colder water distribution and *Ampelisca* occurs generally in warmer, more tropical climates (Dauvin et al. 2021). *Ampelisca* and *Byblis* have the widest depth ranges of the genera ranging from intertidal depths to abyssal regions (Bellan-Santini and Dauvin 2008).

The whole Ampeliscidae family has very conservative morphology. The genera that are the most distinct are *Byblisoides*, distinguished by a short and stubby antenna 1, and *Haploops* which has a different rectangularly shaped pereopod 7 basis. Until comprehensive molecular information on a wider range of species becomes available, the relationships between and internally within the genera will remain speculative. Adding molecular information will eventually allow a deeper understanding of the evolution within the family and the relationships between this family and other amphipods. Here we add two new species to the family Ampeliscidae, one in *Byblis* and one in *Byblisoides*, collected from the Clarion-Clipperton Zone at abyssal depths of 4097–4368 m. We provide descriptions of the new species and keys to the deep-water *Byblis* species and to the *Byblisoides* species of the world. We also provide molecular sequence data for the new taxa.

## Material and methods

### Specific methodology description

The material for the present study was sampled in the central east Pacific, specifically in the easternmost sector of the Clarion-Clipperton Zone (CCZ). The material was collected either by epibenthic sledge (EBS) or boxcore (BC) during six scientific deep-sea cruises: the ABYSSLINE (ABYSSal baseLINE project) cruise AB02 in 2015 to the UKSR-1 and OMS exploration contract areas, four cruises to the NORI-D exploration contract area (C5A, C5D, C7A, and C7B) between 2020 and 2022, and the MANGAN 2023 cruise to the BGR exploration contract area. For details of gear deployment and sample processing see Jażdżewska and Horton (2026).

Individuals were initially examined using a Nikon SMZ800 stereomicroscope. Hand drawings of the habitus were prepared using a Nikon SMZ1500 stereomicroscope equipped with a camera lucida. Pencil drawings from the microscope were used as the basis for line drawings. The drawings were inked by hand and plates composed with Adobe Photoshop CS6.

The habitus of *Byblisoides jazdzewskae* sp. nov. is presented as a photograph obtained with a confocal laser scanning microscope (CLSM). The holotype was stained in Congo red and acid fuchsin, temporarily mounted onto slides with glycerine and examined with a Leica TCS SPV equipped with a Leica DM5000 B upright microscope and three visible-light lasers (DPSS 10 mW 561 nm; HeNe 10 mW 633 nm; Ar 100 mW 458, 476, 488 and 514 nm), combined with the software LAS AF 2.2.1 (Leica Application Suite, Advanced Fluorescence). A series of photographic stacks were obtained, collecting overlapping optical sections throughout the whole preparation (Michels and Büntzow 2010; Kamanli et al. 2017). Chosen specimens were dissected and mounted on permanent slides using polyvinyl-lactophenol containing lignin pink. All slides were examined using a Nikon Eclipse Ci compound microscope equipped with a camera lucida.

In the descriptions and figures the following abbreviations were used: **A1, A2** = antenna 1, 2; **UL** = upper lip; **LL** = lower lip; **Md** = mandible; **R** = right; **L** = left; **Mx1, 2** = maxillae 1, 2; **Mxp** = maxilliped; **G1, G2** = gnathopod 1, 2; **oo** = oostegite; **P3–P7** = pereopods 3–7; **U1–U3** = uropod 1–3; **T** = telson; **Epim** = epimeron; **m** = male; **f** = female.

The registered type material is deposited in the Senckenberg Museum (Frankfurt, Germany) (**SMF**) and the Natural History Museum, London (**NHMUK**). All the remaining material is kept in the Deutsches Zentrum für Marine Biodiversitäts­forschung (**DZMB**) in Wilhelmshaven.

### DNA extraction, amplification, and sequencing

All individuals (14 *Byblis* specimens and six *Byblisoides* specimens) had their cytochrome c oxidase subunit I gene (*COI*) barcoded prior to identification of the species. The molecular procedures for samples collected from the UKSR-1, OMS, and BGR exploration contract areas are described in Jażdżewska et al. (2025).

Specimens from the NORI-D area were processed as follows. DNA was extracted from a pair of pleopods using QuickExtractTM DNA extraction solution (Lucigen), following manufacturer guidelines, and adapted for a digestion time of 45 min. A fragment of the COI genetic marker was amplified using LCO1490 (GGTCAACAAATCATAAAGATATTGG) and HCO2198 (TAAACTTCAGGGTGACCAAAAAATCA) primers (Folmer et al. 1994). A fragment of the 16S rRNA marker was amplified using 16SFt_amp (GCRGTATIYTRACYGTGCTAAGG) and 16SRt_amp (CTGGCTTAAACCGRTYTGAACTC) primers (Lörz et al. 2018). The PCR mix for each reaction contained 10.5 μl of Red Taq DNA Polymerase 1.1X MasterMix (VWR), 0.5 μl of each primer (10 μM), and 1 μl of DNA template. PCR conditions used were an initial denaturation at 95 °C for 5 min, followed by 35 cycles of denaturation at 95 °C for 45 s, annealing at 49 °C for COI and 50 °C for 16S for 45 s, and extension at 72 °C for 1 min, with a final extension of 72 °C for 10 min. The primers used for sequencing were the same as those for amplifications. PCR products were purified using a Millipore Multiscreen 96-well PCR Purification System and sequenced using an ABI 3730XL DNA Analyzer (Applied Biosystems) at The Natural History Museum Sequencing Facilities. For each gene fragment, contigs were assembled by aligning both forward and reverse sequences, chromatograms were visually inspected, and ambiguous base calls were corrected manually, using Geneious 7.0.6 (Kearse et al. 2012).

## Results

### Systematics

**Order Amphipoda Latreille, 1816**

**Suborder Amphilochidea Boeck, 1871**

**Superfamily Synopioidea Dana, 1852**

#### Family Ampeliscidae

Taxon classificationAnimaliaAmphipodaAmpeliscidae

Krøyer, 1842

A7873676-4AAC-5F60-AD61-8ED52BA76B3F

Ampeliscidae Krøyer, 1842: 141–166

##### Type genus.

*Ampelisca* Krøyer, 1842, by original designation.

##### Diagnosis.

Urosomites 2–3 coalesced. Pereopods 5 and 6 alike, basis rhomboid or diamond-shaped but pereopod 7 distinct, basis has distinct shape with extended posterodistal lobe. Eyes can be absent, one or two pairs. When eyes present, composed of internal pigment masses served by 2–4 cuticular lenses. Antenna 1 accessory flagellum absent. Pereopod 3 and 4 merus elongate, propodus much shorter than merus, carpus shorter than propodus. Pereopods 3 and 4 glandular. Head large. Gnathopods weakly subchelate and feeble. Uropod 3 biramous. Telson laminar (after Barnard and Karaman 1991).

##### Genera included.

*Ampelisca* Krøyer, 1842; *Byblis* Boeck, 1871; *Byblisoides* K.H. Barnard, 1931 and *Haploops* Liljeborg, 1856.

##### Remarks.

The family Ampeliscidae includes four genera consisting of 328 species, the most of which belong in the genus *Ampelisca* (210 species). The family has conservative morphology and occurs in all the world’s oceans living in fine sediments on the sea floor at depths ranging from intertidal to abyssal depths ~ 6500 m (*Byblis vitjazi* Margulis, 1967).

#### Genus Byblis

Taxon classificationAnimaliaLamialesByblidaceae

Boeck, 1871

45209128-415F-532B-8541-6E6333B6A05B

Byblis Boeck 1871: 228. —Stebbing 1906: 1211. —Barnard 1966: 55 (key). —Barnard 1969: 130. —Lincoln 1979: 122. —Ledoyer 1982: 82. —Barnard and Karaman 1991: 84–90, figs 22D, 23B. —Bellan-Santini and Dauvin 1993: 909–932. —Ren 2006: 141–199, figs 44–72. —Poore and Lowry 2023: 528–532, figs 6–9.

##### Type species.

*Byblis gaimardii* (Krøyer, 1842) (type by monotypy).

##### Generic diagnosis.

Flagella of antennae 1 and 2 with five or more articles. Article 3 of maxillipedal palp unproduced. Basis of pereopod 7 with a posteroventral margin extended and expanded distally, forming a large lobe; anterior margin of posteroventral lobe with setae lining the margin all the way to the junction between the basis and the ischium. Telson varying from rarely as long as to usually shorter than broad, cleft or incised less than half its length (after Barnard and Karaman 1991; Bellan-Santini and Dauvin 1993).

##### Species.

80 species as listed in Horton et al. (2024) including the proposed new species, *Byblis hortonae* sp. nov.

##### Remarks.

Species of the genus *Byblis* occur in all oceans. Of the 80 species (including the new species proposed here), 62 have been recorded from the Pacific Ocean (Bellan-Santini and Dauvin 1993; Horton et al. 2024). However, four of these species have also been recorded from other oceans and have complex taxonomy, therefore they must be considered questionable distribution records (Bellan-Santini and Dauvin 1993). The majority of these Pacific species are from the western Pacific due to comprehensive studies in Chinese and Vietnamese waters (Ren 1998, 2006) and Eastern Australian waters (Lowry and Poore 1985; Poore and Lowry 2023). Species recorded from the eastern Pacific Ocean are relatively few, including 11 of the 80 species.

*Byblis* species are sediment dwellers occurring from *Globigerina* muds to silty sand, coral rubble, and algae substrates (Bellan-Santini and Dauvin 1993; Poore and Lowry 2023). The species predominantly inhabit shallower waters (65% of species living in shallower than 200 m deep). Only five of the species can be found in truly deep waters, exceeding 2500 m in depth: *Byblis ceylonica* J.L. Barnard, 1961 (3310 m, Indian Ocean, one specimen); *Byblis gloriosae* Ledoyer, 1982 (3700 m, Indian Ocean, three specimens); *Byblis nana* Margulis, 1967 (2795 m, Kuril-Kamchatka Trench, northwestern Pacific Ocean, three specimens); *Byblis vitjazi* Margulis, 1967 (5680–6126 m, Kuril Kamchatka Trench, northwestern Pacific Ocean, four specimens), and the new species, *Byblis hortonae* sp. nov. (4100–4368 m, Clarion-Clipperton Fracture Zone, eastern Pacific Ocean, 16 specimens).

##### Distribution.

Pacific Ocean, Indian Ocean, Atlantic Ocean, Bering Sea, Southern Ocean.

#### Byblis hortonae sp. nov.

Taxon classificationAnimaliaLamialesByblidaceae

D3CD50C5-2CCF-5EA6-982E-1AE1FA0F4BD8

https://zoobank.org/40B3B4B9-71E7-4788-9705-DDC13BECE364

Figs 1, 2, 3, 4, 5

##### Type material.

***Holotype***: • Clarion-Clipperton Zone; 10.3339°N, 117.186°W, 4279 m; 3 December 2022; NORI-D exploration contract area, MV Island Pride, Cruise 7B, Station TF007_04, Box Core BC_481; Specimen: 10326_TH_AMP_1; NHMUK 2025.17; COI (PV077110); 16S (PV077018). ***Paratypes***: Pacific • Clarion-Clipperton Zone; 10.3343°N, 117.177°W, 4282 m; 21 November 2022; NORI-D exploration contract area, MV Island Pride, Cruise 7B, Station TF025_01, Box Core BC_463; Specimen: 9689_TH_AMP_1; NHMUK 2025.18; COI (PV077107). Clarion-Clipperton Zone; 12.3387°N, 116.669°W, 4158 m; 9 March 2020; UKSR-1 exploration contract area, MV Pacific Constructor, Resource Cruise 01 (RC01), station ST56, Box Core BC_28; Specimen: 4082_TH_AMP_1; NHMUK 2025.27. Clarion-Clipperton Zone; 10.3244°N, 117.187°W, 4280 m; 12 May 2021; NORI-D exploration contract area, Maersk Launcher, Cruise 5D, Station STM_180, Box Core BC_389; Specimen: 6881_TH_AMP_1; NHMUK 2025.19; COI (PV077104). Clarion-Clipperton Zone; 10.3289°N, 117.197°W, 4283 m; 11 September 2020; NORI-D exploration contract area, Maersk Launcher, Cruise 5A, Station STM_005, Box Core BC_349; Specimen: 8792_TH_AMP_1; NHMUK 2025.20. Clarion-Clipperton Zone; 10.3552°N, 117.169°W, 4275 m; 27 November 2022; NORI-D exploration contract area, MV Island Pride, Cruise 7B, Station STM_010, Box Core BC_470; Specimen: 10019_TH_AMP_1; NHMUK 2025.21; COI (PV077108); 16S (PV077017). Clarion-Clipperton Zone; 10.3552°N, 117.169°W, 4275 m; 27 November 2022; NORI-D exploration contract area, MV Island Pride, Cruise 7B, Station STM_010, Box Core BC_470; Specimen: 10020_TH_AMP_1; NHMUK 2025.22; COI (PV077109). Clarion-Clipperton Zone; 10.3343°N, 117.177°W, 4282 m; 21 November 2022; NORI-D exploration contract area, MV Island Pride, Cruise 7B, Station TF025_01, Box Core BC_463; Specimen: 10378_TH_AMP_1; NHMUK 2025.23; COI (PV077111); 16S (PV077016). Clarion-Clipperton Zone; 10.3303°N, 117.175°W, 4285 m; 26 August 2022; NORI-D exploration contract area, MV Island Pride, Cruise 7A, Station TF_027, Box Core BC_431; Specimen: 9344_TH_AMP_1; NHMUK 2025.24; COI (PV077106); 16S (PV077016). Clarion-Clipperton Zone; 10.3342°N, 117.177°W, 4284 m; 27 August 2022; NORI-D exploration contract area, MV Island Pride, Cruise 7A, Station TF_027, Box Core BC_435; Specimen: 9315_TH_AMP_1; NHMUK 2025.25; COI (PV077105).

**Figure 1.**
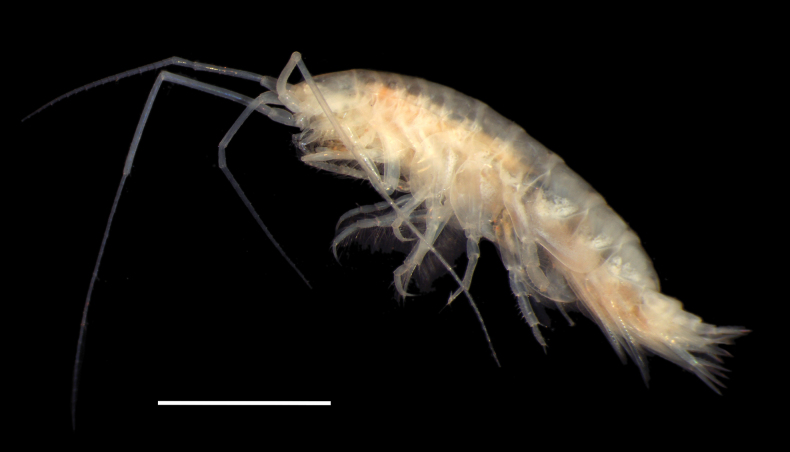
*Byblis hortonae* sp. nov., paratype, specimen 10019_TH_AMP_1, NHMUK 2025.18, male, 14 mm. Scale bar: 5 mm.

**Figure 2.**
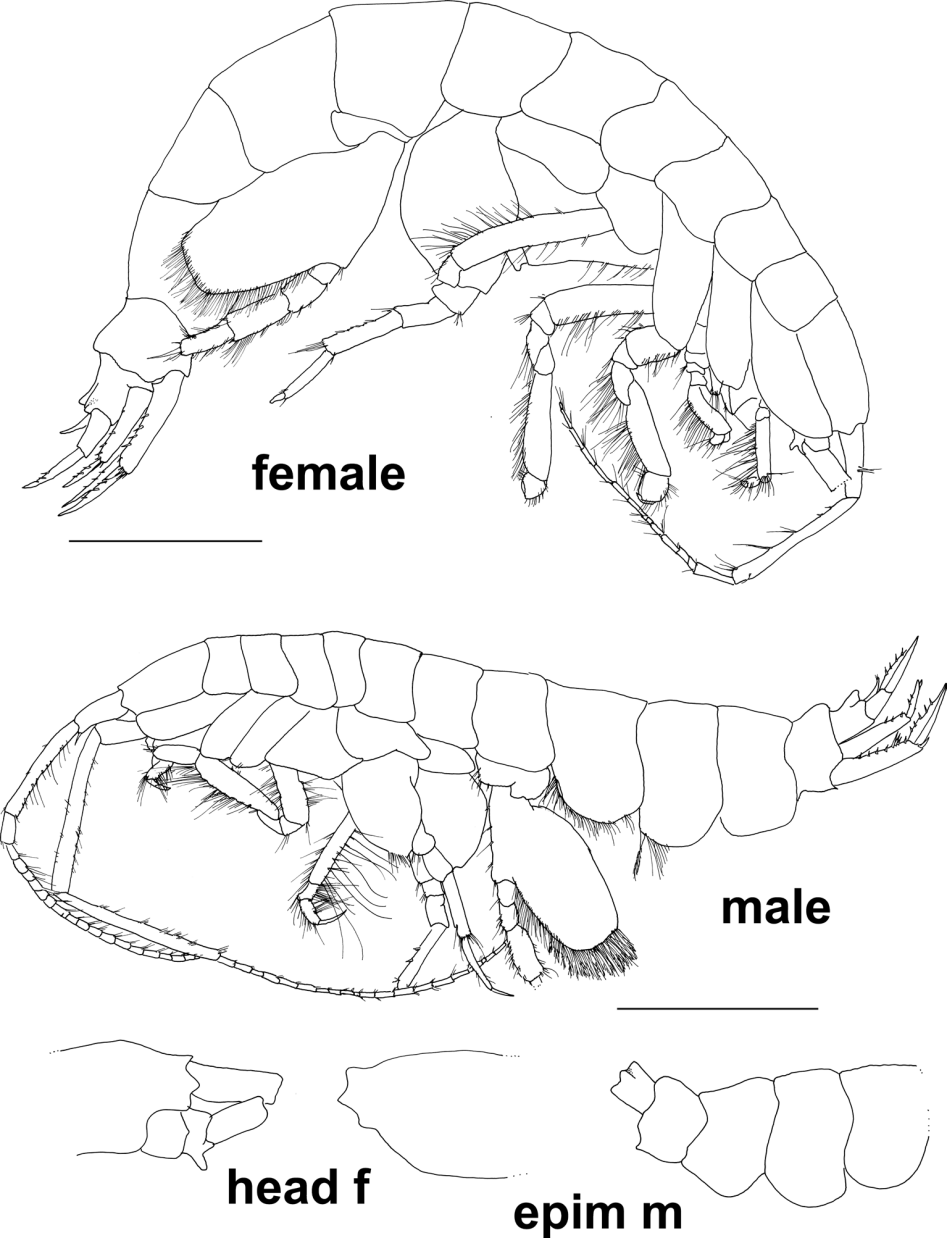
*Byblis hortonae* sp. nov., female, holotype, 16 mm, #10326, NHMUK 2025.17, whole animal, head; paratype, male, 17 mm, #10019, NHMUK 2025.18, whole animal, epimeron. Scale bars: 0.5 mm.

**Figure 3.**
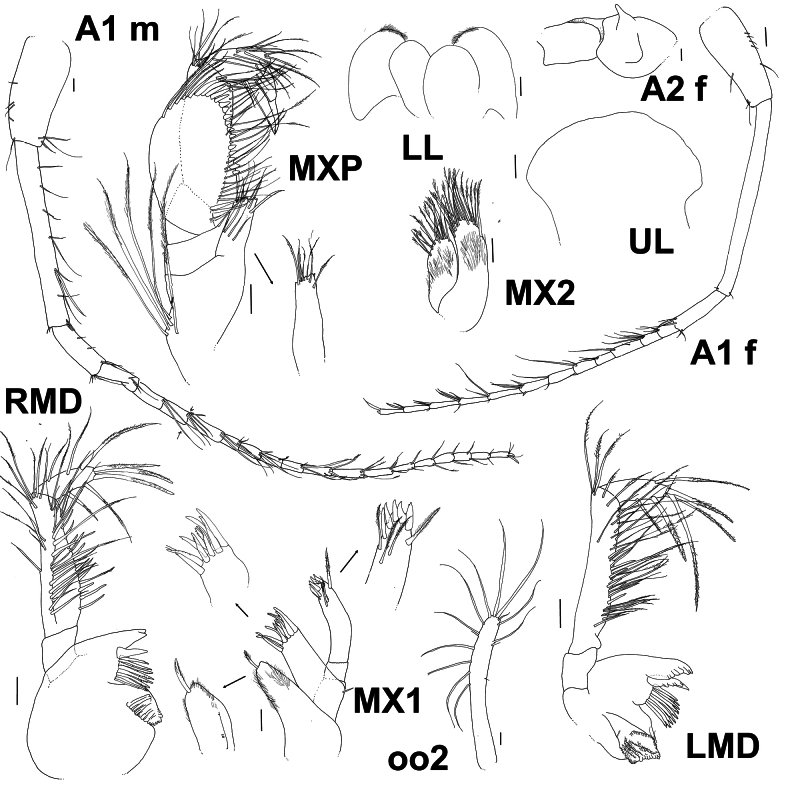
*Byblis hortonae* sp. nov. holotype female, A1 and A2, mouthparts. Paratype male, A1 and A2. Scale bars: 0.1 mm.

**Figure 4.**
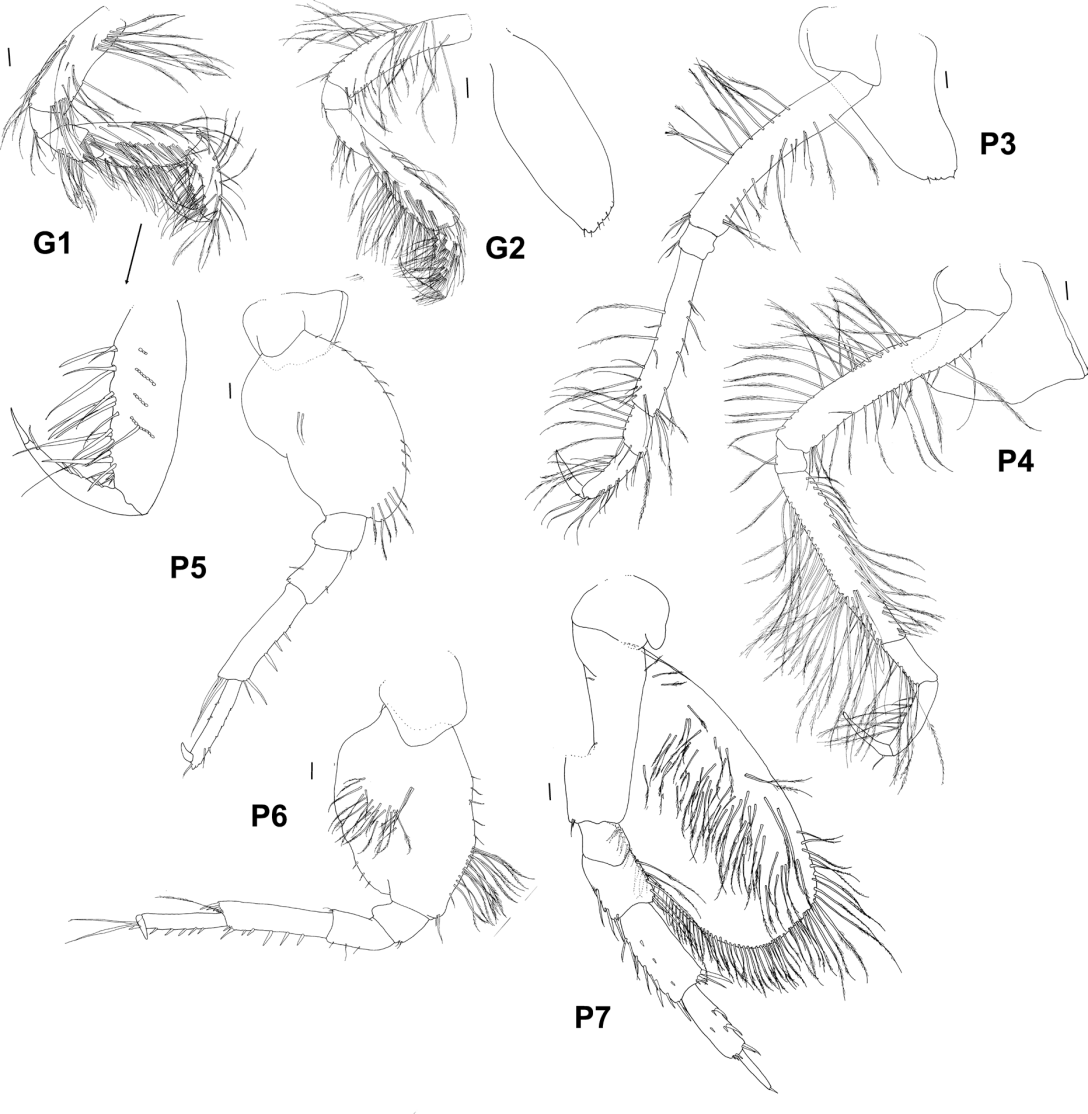
*Byblis hortonae* sp. nov. holotype female, G1 and G2, P3–P7. Paratype male, G1 and G2. Scale bars: 0.1 mm.

**Figure 5.**
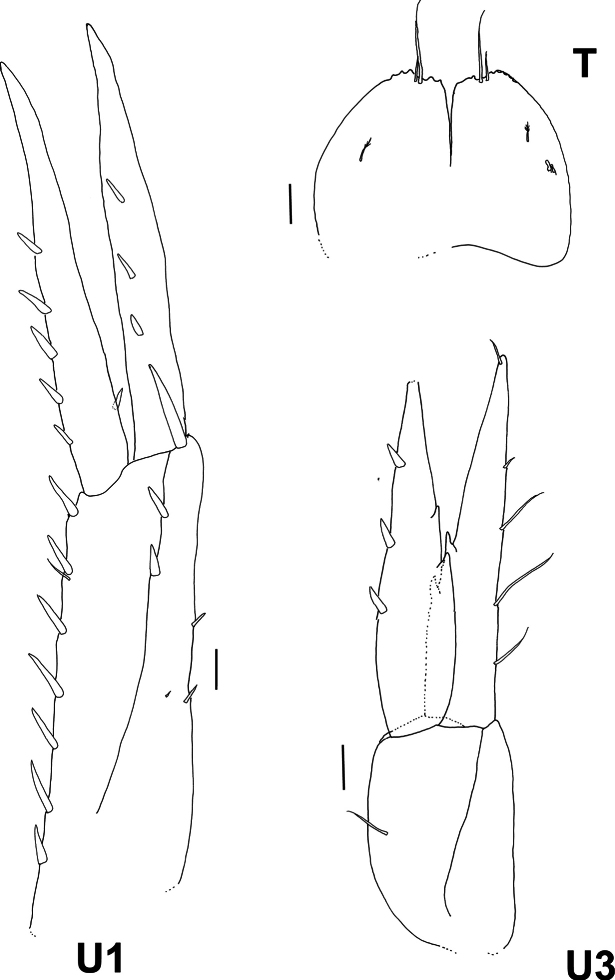
*Byblis hortonae* sp. nov. holotype female, U1, 3, T. Scale bars: 0.1 mm.

##### Other material.

Clarion-Clipperton Zone; 11.9331°N, 116.2451°W–11.9332°N, 116.222°W, 4150-4151 m; 8 May 2018; BGR exploration contract area, RV Sonne, Cruise SO262, Station SO 262-151, Epibenthic Sledge; Specimen: DSB_3543; COI (PQ734224). Clarion-Clipperton Zone; 12.531°N, 116.623°W, 4219 m; 20 February 2015; UKSR-1 exploration contract area, RV Thompson, Cruise AB02, Station AB2-EB02, Epibenthic Sledge; Specimen: DSB_3745; COI (PQ734760). Clarion-Clipperton Zone; 12.531°N, 116.623°W, 4219 m; 20 February 2015; UKSR-1 exploration contract area, RV Thompson, Cruise AB02, Station AB2-EB02, Epibenthic Sledge; Specimen: DSB_3750; COI (PQ734591). Clarion-Clipperton Zone; 12.102°N, 117.181°W, 4100 m; 26 February 2015; OMS exploration contract area, RV Thompson, Cruise AB02, Station AB2-EB05, Epibenthic Sledge; Specimen: DSB_3768; COI (PQ734278). Clarion-Clipperton Zone; 12.503°N, 116.509°W, 4244 m; 4 March 2015; UKRS-1 exploration contract area, RV Thompson, Cruise AB02, Station AB2-EB08, Epibenthic Sledge; Specimen: DSB_3788; COI (PQ734446). Clarion-Clipperton Zone; 11.6042°N, 118.0497°W–11.6184°N, 118.0205°W, 4368-4356 m; 4 May 2023; BGR exploration contract area, RV Kilo Moana, Station KM23-69, Epibenthic Sledge; Specimen: DSB_8388; COI (PQ734385).

##### Type locality.

Clarion-Clipperton Zone, 10.3339°N, 117.186°W, 4279 m.

##### Etymology.

This species is named for Dr Tammy Horton in recognition of her extensive work on and love for amphipods.

##### Diagnosis.

Head longer than deep. Anterolateral corner of the head not produced. Eyes and cuticular lenses absent. Coxa 1 reaching anterior margin of the head. Antenna 1 shorter than antenna 2, reaching past the distal end of antenna 2 peduncle. Antenna 2 reaching ~ 3/4 body length, article 4 of peduncle subequal in length to article 5. Gnathopods 1 and 2 weakly subchelate, palm strongly acute. Pereopod 7 basis posterior margin extended obliquely to form an expanded posterodistal lobe; anterior margin of basis setose near the junction of the basis and ischium; dactylus spine-like; basis posterodistal lobe broadly rounded, reaching to the length of the distal margin of the carpus. Telson lobes with two pairs of apical slender setae, no robust setae; wider than long; telsonic lobes apically truncated at a slight angle and weakly serrate.

##### Description.

Based on holotype, female, 16 mm, 10326_TH_AMP1, NHMUK 2025.17.

***Body***: (Figs 1, 2). ***Pereon*** slender and strongly laterally compressed. Oostegites long and narrow bearing long slender slightly curved setae. ***Epimera*** all narrowly rounded ventrally, epimeron 1 and 2 setose along ventral margins, epimeron 3 without setae. ***Urosome*** urosomite 1 slightly raised dorsally to form a subacute tooth, urosomites 2 and 3 produced to form a strongly produced subacute carina.

***Head***: (Figs 1, 2, 3). ***Head*** 1.5× as long as deep. Rostrum very small, 0.07× head length and blunt, anteroventral margin with small antennal lobe, same length as rostrum, ventral margin oblique. ***Antenna 1*** peduncle article 1 0.5× article 2, peduncular article 2 3.3× article 3, peduncular article 3 0.6× article 1, flagellum 12-articulate. ***Antenna 2*** broken off in holotype, only articles 1–3 of peduncle present, strongly prominent gland cone present on article 2.

***Mouthparts***: (Fig. 3) ***Upper Lip*** broad, setae absent. ***Left mandible*** molar strongly triturating, with nine accessory toothed robust setae, lacinia mobilis 5-toothed, incisor with eight teeth, palp long and robust, article 1 0.2× article 2, without setae, article 2 2.6× article 3, lined along anterior and distal margins with many long slender slightly plumose setae, proximal end of article 2 with small lobe at junction with article 1, article 3 1.9× article 1, margins bearing six long robust slightly plumose setae, article 3 almost straight sided. ***Right mandible*** molar strongly triturating, with nine accessory toothed robust setae, lacinia mobilis obscured, incisor with two teeth, palp long and robust, article 1 0.3× article 2, without setae, article 2 2.5× article 3, lined along anterior and distal margins with many long slender slightly plumose setae, article 3 1.4× article 1, margins bearing six long robust slightly plumose setae, article 3 almost straight sided. ***Lower lip*** broad, with light fringe of setae. ***Maxilla 1*** inner plate nearly reaching the length of the outer plate, covered in fine setae apically with one long plumose slender to robust seta, outer plate with nine long toothed robust setae, palp bi-articulate, article 2 tipped with five strong, curved robust setae and submarginally with four long plumose setae. ***Maxilla 2*** inner plate one-third of the length of outer plate, similar widths, both plates covered in fine setae and tipped with many long, plumose slender setae. ***Maxilliped*** inner plate reaching one-third along outer plate, apically truncated and weakly serrated, with seven long slender setae line on the apical margin, outer plate ovoid, inner margin lined with robust setae, triangular setae proximally gradually becoming longer, narrower and plumose distally, palp 4-articulate, article 2 narrow, inner margin lined with long, slender plumose setae, article 3 ovoid and not forming a hood around article 4 and lined with long, slender, plumose setae, article 4 slender, slightly longer (including the nail) than article 3.

***Pereon***: (Figs 2, 4) ***Gnathopod 1*** coxa extended reaching to the anterolateral margin of the head, ventral margin weakly serrate lined with long slender plumose setae, basis narrow, lined with long, slender, plumose setae, basis and carpus subequal in length and width, ischium 0.43× merus, merus 0.4× carpus, lined with long, slender, plumose setae, carpus subrectangular and heavily lined with long, slender, plumose setae, 1.5× length of propodus, propodus narrowly ovoid, lined with strong, slightly plumose setae, 1.2× dactylus, weakly subchelate, palm acute, palm defined by long, strong slender – robust setae of varying lengths, dactylus curving gently, inner margin lined with four short, narrow robust setae. ***Gnathopod 2*** similar in size, shape, setation and chelation to gnathopod 1, basis subequal in length to carpus, ischium 0.44× merus, merus 0.4× carpus, carpus 2.0× propodus, propodus 1.25× dactylus, dactylus inner margin with four narrow robust setae. ***Pereopods 3 and 4*** dactylus shorter than propodus. ***Pereopod 5*** basis relatively long and narrow compared to other ampeliscids (length 1.4× width), posterior margin without setae, sinuous in shape, anterior margin evenly rounded, lined with short slender setae, anteroventral corner bearing six long, slender plumose setae, basis 4.7× ischium, ischium with only two small slender setae on the anteroventral corner, 0.7× merus, merus rectangular with only one slender seta on each margin, 0.5× carpus, carpus long and rectangular, 1.2× propodus, anterior margin bearing four narrow robust setae, posterodistal corner bearing a cluster of four setae (two robust and two slender), propodus rectangular, 4.5× dactylus, with small slender setae lining each margin, anterodistal corner extended to form a slightly serrated subacute lobe bearing two slender setae, lobe reaching past insertion point of dactylus. 0.3× length of dactylus, dactylus slightly curved. ***Pereopod 6*** basis relatively long and narrow (length 1.35× width), 5.2× ischium, posterior margin with three distal small slender setae, evenly curved shape, anterior margin unevenly rounded proximal half lined with short slender setae, distal half lined with long slender plumose setae, anteroventral corner bearing two short, slender plumose setae, medial face with large patch of long, slender plumose setae, ischium with only two small slender setae on the anteroventral corner, 0.6× merus, merus rectangular with slender seta on both margins, 0.5× carpus, carpus long and rectangular, 1.3× propodus, anterior margin bearing five narrow robust setae, posterodistal corner bearing a row of five robust setae increasing in length and one long slender plumose seta, propodus rectangular, 3.4× dactylus, anterior margin lined with six small robust, anterodistal corner extended to form a slightly serrated subacute lobe bearing two long slender setae, lobe reaching past insertion point of dactylus. 0.3× length of dactylus, dactylus slightly curved. ***Pereopod 7*** basis extended to form a long narrow rounded posteroventral lobe, lobe past the basis junction with the ischium subequal in length to anterior margin of basis and reaching to the distal margin of the carpus, anterior margin of main basis bearing a few slender simple setae, posteroventral lobe margin lined with long, slender, plumose setae, reaching all along the anterior margin of the lobe to the junction of the basis to the ischium, medial face of lobe covered in long slender, plumose setae, basis (without lobe) 3.9× ischium, ischium without setae, 0.9× merus, merus rectangular with five long, slender, plumose setae on posterior margin and three long slender setae on anterior margin, 0.5× carpus, carpus long and rectangular, 1.2× propodus, anterior margin bearing eight long narrow robust setae, posterodistal corner bearing a cluster of four robust setae of increasing size, medial face with line of three small robust setae, propodus rectangular, 1.5× dactylus, posterior margin with two clusters of two robust setae along margin and two small robust setae on each distal corner, anterior margin without setae, dactylus long, narrow and straight-sided.

***Urosome***: (Fig. 5) ***Uropod 1*** rami subequal in length, peduncle slightly longer than rami, dorsal margin bearing row of seven long robust setae, ventral margin bearing three small robust setae, medial margin with two large robust setae, distoventral corner bearing large curved robust seta, outer ramus bearing three medial robust setae, inner ramus bearing five robust setae on outer margin. ***Uropod 2*** peduncle longer than rami, rami subequal in length, peduncle with four large robust setae on each margin, outer ramus bearing two robust setae on the medial face, inner ramus bearing two large robust setae on outer margin. ***Uropod 3*** peduncle shorter than rami, bearing only one slender seta, rami subequal, outer ramus outer margin bearing four long slender simple setae, inner margin without setae but with two large mid-marginal teeth, and tipped with a small slender seta, inner ramus outer margin bearing three large robust setae, inner margin without setae but bearing two large mid-marginal teeth, tip broken off. ***Telson*** 1.3× wider than long, cleft to half of length, each lobe truncated and weakly serrate apically, each lobe bearing two slender simple setae, one long and one short, one short plumose sensory seta on each lobe on the mid-medial face, and two clasping hooks on each lobe.

##### Variation.

Based on paratype, male, 14 mm, 10019_TH_AMP1, NHMUK 2025.17.

***Body***: (Fig. 2) ***Pleon*** epimera 1 and 2 strongly setose on ventral margin, including plumose setae, epimeron 3 narrowly rounded to subquadrate, without setae. ***Urosome*** urosomite 1 strongly produced to form a distinct keel and rounded corner, urosomites 2 and 3 produced to form a narrowly rounded carina (Fig. 2).

***Head*: *Antenna 1*** reaching just past antenna 2 peduncle, peduncular article 1 0.56× article 2, article 2 3.2× article 3, article 0.6× article 1, flagellum with 17-articulate, distal article tipped with two long narrow robust setae. ***Antenna 2*** reaching to epimeron 2.

***Urosome*: *Uropod 3*** peduncle shorter than rami, peduncle with three slender and one robust seta, rami of subequal length, outer ramus outer margin bearing six long slender setae, inner margin with two slender setae and five strong teeth and tipped with on slender seta, inner ramus outer margin with five strong robust setae and inner margin without setae but with five smallish but prominent teeth.

##### Remarks.

As mentioned in Myers (2012) and Poore and Lowry (2023) the morphology of species in the genus *Byblis* is very conservative, stating “*Byblis* species are rather uniform in design, with character states being found in myriad combinations. This makes it difficult to assign *Byblis* species to groups and therefore difficult to compare new species.” (Myers 2012: 5; Poore and Lowry 2023: 528). There are relatively few *Byblis* species occurring in deep waters and even fewer from deep waters in the Pacific Ocean (see below key). These are *B. gerara* Lowry & Poore, 1985, *B. vitjazi* Margulis, 1967, *B. nana* Margulis, 1967, *B. coeca* Margulis, 1967, and the newly proposed *Byblis hortonae* sp. nov. The new species can be distinguished from the aforementioned species particularly as follows: by the prominence and angle of the antenna 2 gland cone which is directed almost directly perpendicular to article 2 of the peduncle (reduced and angled anteriorly in the other four species) and the relative lengths of antenna 1 to antenna 2. In the new species, antenna 1 reaches just to the end of the peduncle of antenna 2 whereas in *B. gerara* it is distinctly shorter than the end of the peduncle of antenna 2 and is as long as the whole antenna 2 (including flagellum) in the other three species. The new species also differs from the other Pacific deep species by the shape and setation of the telson. The new species has the telson wider than long, as does *B. nana* (*B. gerara* is distinctly longer than wide, and *B. vitjazi* and *B. coeca* have the telson as long as wide), and the telson has a distinctly rectangular profile with the tips of the lobes mostly truncated, slightly angled inward with two slender setae and weak serrations apically on each lobe (*B. gerara*, *B. vitjazi*, and *B. coeca* all have a smooth triangular shaped telson). *Byblis nana* has a similar shaped telson to the new species but not quite as truncated, more rounded, without the serrations and only one robust seta apically. A summary of the differences between the deep sea Pacific *Byblis* species diagnoses is provided (Table 1). This combination of characters, geography, and depth places this species as different from those already known to science. Two separate collections were obtained for this species, and both were sequenced separately. The sequences align to each other indicating this species is distributed throughout the Clarion-Clipperton Zone.

**Table 1.** Morphological comparison of deep sea Pacific *Byblis* species.

	***B. coeca***	***B. gerara***	***B. hortonae* sp. nov**.	***B. nana***	***B. vitjazi***
Head proportion	Longer than deep	Longer than deep	Longer than deep	As long as deep	Longer than deep
Head anteroventral corner	Not produced	Not produced	Not produced	Not produced	Produced
Antenna 1: Antenna 2	A1 nearly = A2	A1 < A2	A1 < A2, reaching past distal end of peduncle	A1 = A2	A1 almost = A2
Antenna 2: body	undocumented	Longer than body	¾ body	½ body	undocumented
Antenna 2 article 4 : article 5	Art 4 < art 5	Art 4 subequal art 5	Art. 4 subequal to art. 5	Art 4 > art 5	Art 4 < art 5
Gnathopods 1 and 2 chelation	Weakly subchelate	Strongly subchelate	Weakly subchelate	Weakly subchelate	Weakly subchelate
Gnathopods 1 and 2 palm angles	Strongly acute	Weakly acute, almost transverse	Strongly acute	Strongly acute	Strongly acute
Gnathopods 1 and 2 carpi to propodus widths	Carpus similar width to propodus	Carpus wider than propodus	Carpus similar width to propodus	Carpus similar width to propodus	Carpus wider than propodus
Pereopod 7 basis posterior margin angle	Almost perpendicular to article join	Oblique angle	Oblique angle	Oblique angle	Almost perpendicular to article join
Pereopod 7 basis posterodistal lobe shape	Narrowly rounded, slightly truncated on posterior margin	Broadly rounded	Broadly rounded	Broadly rounded	Broad, slightly flattened posteriorly
Pereopod 7 basis lobe extension	Reaching nearly to the distal margin of carpus	Reaching 1/2 length of merus	Reaching to distal end of carpus	Reaching junction of merus and carpus	Reaching midpoint of carpus
Telson apical setation	One small, slender seta per lobe	One robust and one small seta per lobe, two robust setae medially on each lobe	Two pairs slender setae, no robust setae	One robust seta per lobe	No setae mentioned
Telson proportion	Longer than wide	Longer than wide	Wider than long	Slightly wider than long	Slightly wider than long
Telsonic lobes apical shape	Slightly angled with a distal notch	Narrowly subacute apically	Apically truncated, weakly serrate	Rounded, smooth apically	Apically angled and irregularly serrate

##### Distribution.

Abyssal Pacific Ocean, Clarion-Clipperton Zone, 4100–4368 m.

##### Molecular data.

Sequence data for the holotype of *B. hortonae* sp. nov. is deposited in GenBank under accession number PV077110 (COI) and PV077018 (16S). Sequences of the paratypes are deposited in GenBank with the following accession numbers: PV077104–PV077111 (COI), and PV077016–PV077019 (16S). The species has also received a Barcode Index Number from Barcode of Life Data Systems: BOLD:AEB1213 (https://doi.org/10.5883/BOLD:AEB1213).

### Key to deep sea *Byblis*

Whole occurrence range deeper than 1000 m. All species noted have no eyes or cuticular lenses. (*Byblis cubensis* omitted due to unavailability of the literature).

**Table:** 

1	Anteroventral corner of head produced	*B. vitjazi* Margulis, 1967 ^*^
–	Anteroventral corner of head not produced	**2**
2	Antenna 1 subequal to or longer than antenna 2	**3**
–	Antenna 1 distinctly shorter than antenna 2, reaching to the end of the peduncle or not even that far	**6**
3	Telson as long as wide or longer than wide	**4**
–	Telson wider than long	*B. nana* Margulis, 1967 ^*^
4	Gnathopod 1 propodus and carpus obviously expanded/inflated	***B. gloriosae* Ledoyer, 1982**
–	Gnathopod 1 carpus relatively straight sided	**5**
5	Robust seta on each lobe of telson situated apically	*B. coeca* Margulis, 1967 ^*^
–	Robust seta on each lobe of telson situated medially and half-way down the lobe	***B. bathycephala* Mills, 1971**
6	Telson as long as wide or longer than wide	**7**
–	Telson distinctly wider than long	*B. hortonae* sp. nov. ^*^
7	Gnathopod 1 subchelate with an almost transverse palm	*B. gerara* Lowry & Poore, 1985 ^*^
–	Gnathopod 1 weakly subchelate with a strongly oblique palm	**8**
8	Gnathopod 1 and 2 carpi expanded to give a robust look, telson cleft a quarter the length of telson	***B. ceylonica* J.L. Barnard, 1961**
–	Gnathopod 1 and 2 carpi linear and narrow, telson cleft half the length of telson	***B. medialis* Mills, 1971**

#### Genus Byblisoides

Taxon classificationAnimaliaAmphipodaAmpeliscidae

K.H. Barnard, 1931

3C17A1B5-1A21-548B-A154-C084A13DF8AE

Byblisoides K.H. Barnard 1931: 426.—Mills 1971: 373.—J.L. Barnard and Karaman 1991: 90. —Peart 2018a: 150–157, figs 1–4. —Peart 2018b: 348–363, figs 1–10. —Poore and Lowry 2023: 533.

##### Type species.

*Byblisoides juxtacornis* K.H. Barnard, 1931 (type by original designation).

##### Generic diagnosis.

Flagellum of antennae 1 and 2 with more than or equal to four articles. Maxilliped palp article 3 not distally produced. Pereopod 7 basis posterior margin angled and expanded ventrally, anterior margin of lobe (close to junction with ischium) setose (usually). Telson longer than broad, deeply cleft. Supplementary characters: head long and generally parallel sided. Head anteroventral corner produced (except in *Byblisoides profundi* Mills, 1971). Antenna 1 small, clubbed shaped, not reaching past peduncle of antenna 2. Antenna 2 extended and enlarged, peduncle and flagellum bearing long glass-like setae. Gnathopods reduced and setose. Urosomite 1 weakly carinate (except *B. esferis* J.L. Barnard, 1961).

##### Species composition.

Ten species including the proposed new species: *Byblisoides arcillis* J.L. Barnard, 1961, *B. bellansantiniae* Peart, 2018, *B. blasensis* J.L. Barnard, 1964, *B. esferis* J.L. Barnard, 1961, *B. juxtacornis* K.H. Barnard, 1931, *B. monicae* Peart, 2018, *B. plumicornis* Ledoyer, 1978, *B. profundi* Mills, 1971, *B. richardi* Peart, 2018, *B. jazdzewskae* sp. nov.

##### Remarks.

*Byblisoides* has the least number of species among the ampeliscid genera, with ten species (including the proposed new species). The species in this genus are generally specialized deep-water animals, having been recorded down to depths of more than 4000 m (*Byblisoides profundi* from 4977 m). The genus is wide-ranging, extending from Antarctic waters (*B. juxtacornis* and *B. monicae*), to the Makassar Strait in the western Pacific (*B. arcillis*), to the Tasman Sea, south Pacific Ocean (*B. esferis*), and the Chatham Rise, east of New Zealand, southwestern Pacific Ocean (*B. richardi*), north of the Caribbean, Panama (*B. blasensis*), the Gay Head-Bermuda transect, North American Basin, Atlantic Ocean (*B. profundi*), Madagascar, Indian Ocean (*B. plumicornis*) and Icelandic waters, in the northern Atlantic Ocean (*B. bellansantiniae*). Even though the genus is widespread, each species is usually only known from a single or a few specimens, as is often the case with deep-water taxa.

##### Distribution.

Just more than half of the *Byblisoides* fauna occurs in the Pacific Ocean, occurring at depths of 400–4200 m, including two from Antarctic waters, two from around New Zealand, one from tropical western Pacific, and only one from the eastern Pacific Ocean. There are three species from the Atlantic Ocean and one from the Indian Ocean.

#### Byblisoides jazdzewskae sp. nov.

Taxon classificationAnimaliaAmphipodaAmpeliscidae

20169972-7B05-5A15-BCAA-3DA09D737E35

https://zoobank.org/E2F9D31D-2565-4D17-B32D-0EC6C7BACC8E

Figs 6, 7, 8, 9

##### Type material.

***Holotype***: PACIFIC; female, 15 mm, • Clarion-Clipperton Zone; 12.251°N, 117.32°W, 4137 m; 01 March 2015; OMS exploration contract area, RV Thompson, ABYSSLINE-2 Cruise, Station AB2-EB06, Epibenthic sledge; SMF 63346; COI (PQ734335). ***Paratypes***: young female/male, 13 mm, • Clarion-Clipperton Zone; 12.7.8°N, 117.18.66°W, 4111 m; 25 February 2015; OMS exploration contract area, RV Thompson, ABYSSLINE-2 Cruise, Station AB2-EB04, Epibenthic sledge; Specimen: SMF 63345; COI (PQ734288).

**Figure 6.**
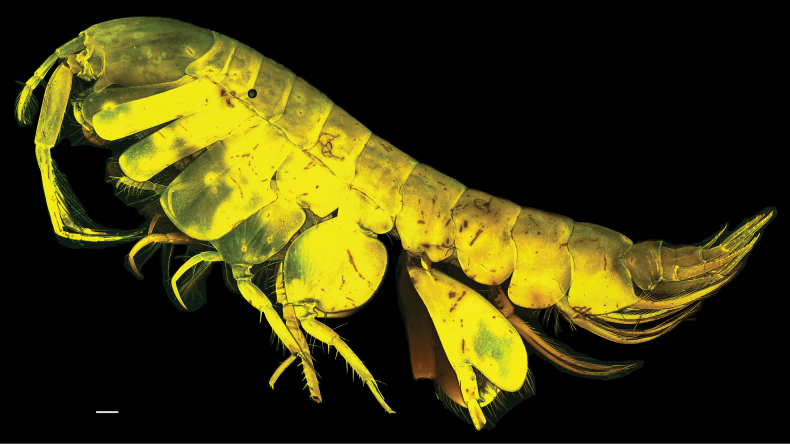
*Byblisoides jazdzewskae* sp. nov. female, holotype, SMF63346 CLSM image. Scale bar: 0.5 mm.

**Figure 7.**
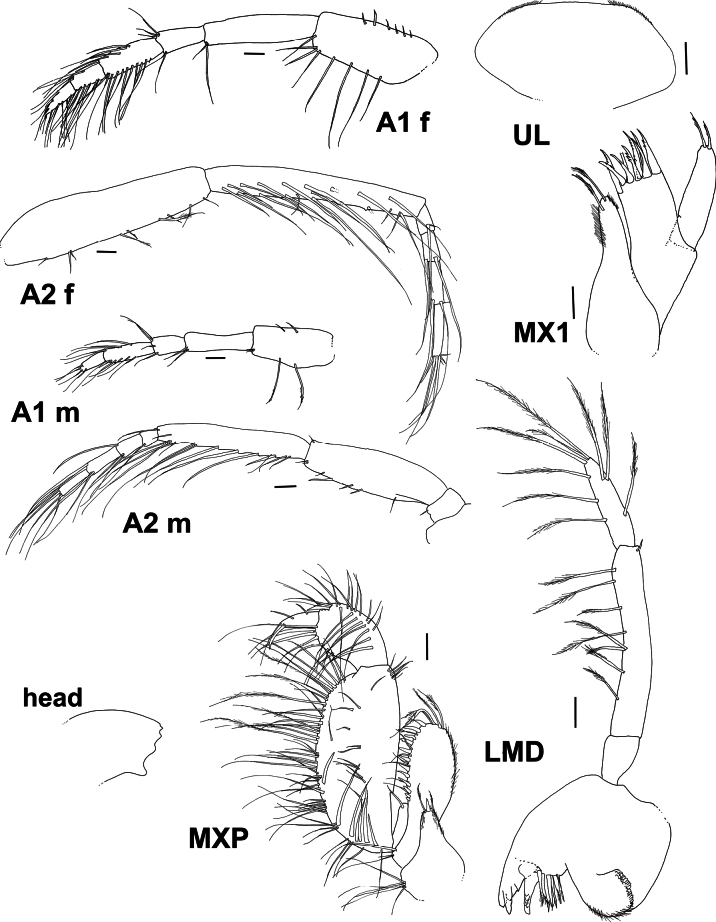
*Byblisoides jazdzewskae* sp. nov. holotype, female, SMF63346, mouthparts, A1 and A2. Paratype, young female/male (m), SMF63345, A1 and A2. Scale bars: 0.1 mm.

**Figure 8.**
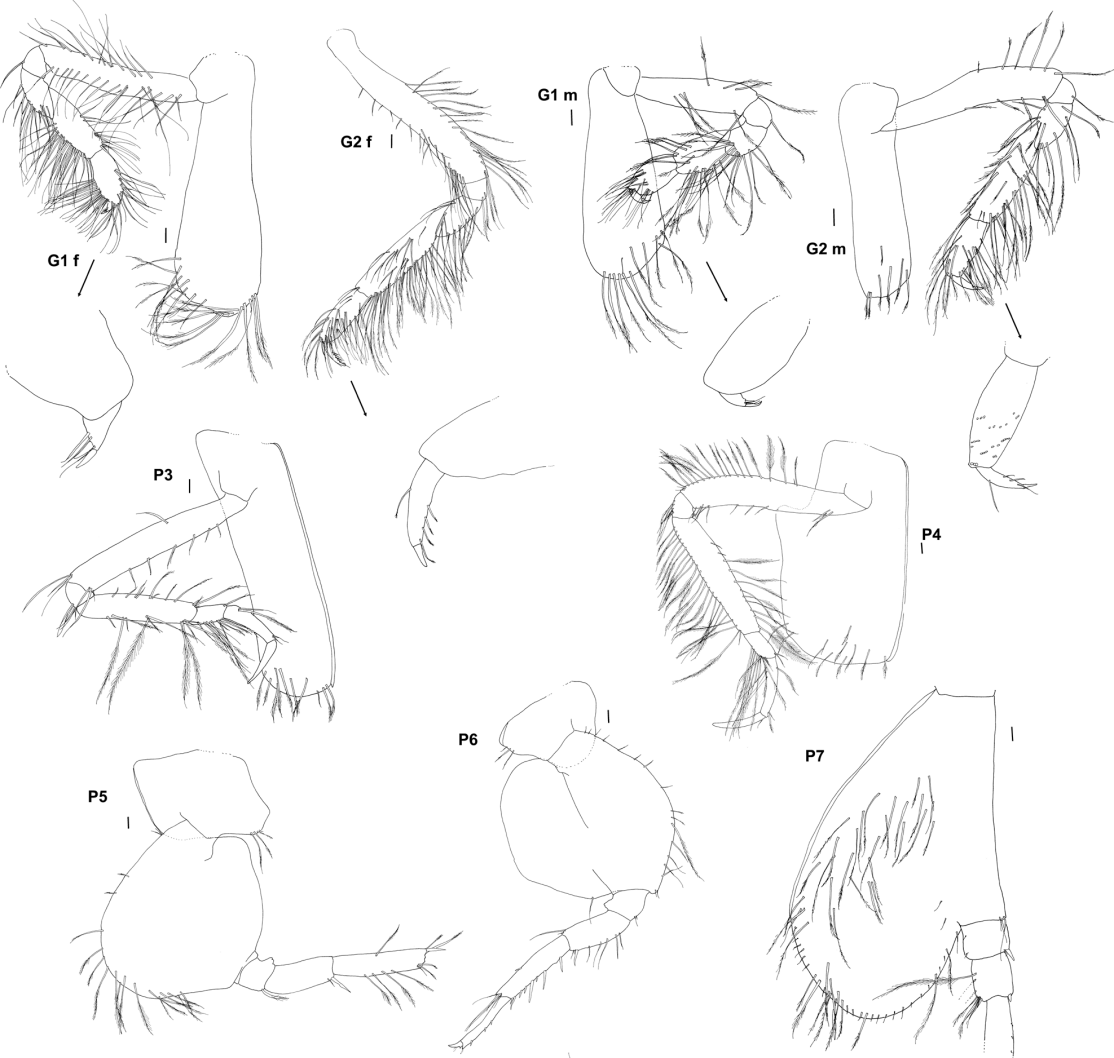
*Byblisoides jazdzewskae* sp. nov. holotype, female, SMF63346, G1 and G2, P3–P7. Paratype, young female/male (m), SMF63345, A1 and A2. Scale bars: 0.1 mm.

**Figure 9.**
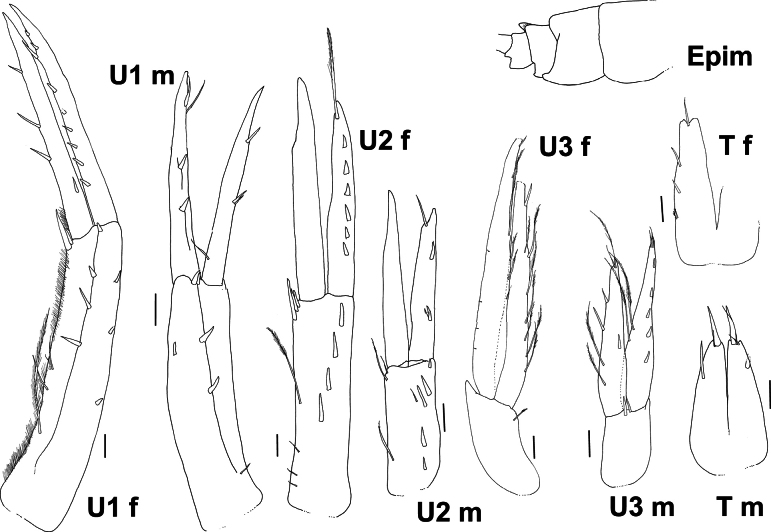
*Byblisoides jazdzewskae* sp. nov. holotype, female, SMF63346, U1–U3, T. Paratype, young female/male (m), SMF63345, A1 and A2. Scale bars: 0.1 mm.

##### Other material.

Clarion-Clipperton Zone; 12.367°N, 116.55°W, 4209 m; 18 February 2015; UKSR-1 exploration contract area, RV Thompson, ABYSSLINE-2 Cruise, Station AB2-EB01, Epibenthic Sledge; Specimen: DSB_3737; COI (PQ734692). Clarion-Clipperton Zone; 12.102°N, 117.181°W, 4100 m; 26 February 2015; OMS exploration contract area, RV Thompson, ABYSSLINE-2 Cruise, Station AB2-EB05, Epibenthic Sledge; Specimen: DSB_3767; COI (PQ734699). Clarion-Clipperton Zone; 12.102°N, 117.181°W, 4100 m; 26 February 2015; OMS exploration contract area, RV Thompson, ABYSSLINE-2 Cruise, Station AB2-EB05, Epibenthic Sledge; Specimen: DSB_3771; COI (PQ734216). Clarion-Clipperton Zone; 11.2965°N, 116.3144°W–11.3091°N, 116.2946°W, 4185-4182 m; 1 May 2023; BGR exploration contract area, RV Kilo Moana, Station KM23-50, Epibenthic Sledge; Specimen: DSB_7940; COI (PQ734707).

##### Type locality.

Clarion-Clipperton Zone, 12.251°N, 117.32°W, 4137 m, Pacific Ocean.

##### Etymology.

This species is named for Dr Anna Jażdżewska for organising and running a wonderful deep-sea amphipod taxonomy workshop where this species was initially described.

##### Diagnosis.

Anteroventral corner of head rounded, produced to slightly exceed the length of the anterior margin of head. Antenna 1 short, reaching to a halfway along antenna 2 peduncle article 4, flagellum 4-articulate. Coxa 1 ventral margin angled anteriorly, uneven. Maxilla 1 palp with only one article. Pereopod 5 basis posterior margin even. Pereopod 7 carpus anterior margin without plumose setae. Pereopod 7 basis posteroventral corner rounded. Uropod 2 inner ramus without marginal robust setae.

##### Description.

Based on holotype, female, 15 mm length, SMF63346.

***Head*** (Figs 6, 9) anteroventral corner produced forward, slightly exceeding level with anterodorsal corner, anterior margin, excavate at antenna 2 insertion, antennal lobe concave, with two small acute points, rostrum absent, head longer than deep, ventral margin straight. *Antenna 1* (Figs 6, 7) short, reaching to halfway along the length of antenna 2 peduncle article 4; peduncle article 1 subequal in length to article 2 (0.95×), article 2 longer than article 3 (2.6×), article 3 shorter than article 1 (0.4×); flagellum shorter than peduncle 3-articulate (article 1 longest), ventral margin of peduncle article 1 and both margins of flagellum with long plumose, slender setae. *Antenna 2* (Figs 6, 7) comparatively stout, reaching to just under half of body length; 1.8× length of head; peduncular article 4 subequal in length to article 5, article 5 ventral and dorsal margin smooth, with long slender setae; flagellum half the length of peduncle article 4 length, 4-articulate.

***Mouthparts***: (Fig. 7) *Upper lip* broad, tipped with two patches of short fine setae. *Left mandible* molar well-developed, weakly triturating, setose, with five toothed and three plumose setae in accessory setal row; incisor toothed with four teeth; lacinia mobilis with five teeth; palp long, article 1 shorter than article 2 (0.26×), article 2 longer than article 3 (1.9×), slightly curved inner margin lined with eight long slender plumose setae, article 3 longer than article 1 (2×), inner margin smooth, with sparse long slender plumose setae. *Right Mandible* molar well-developed, weakly triturating, setose, with seven toothed and six plumose setae in accessory setal row; incisor toothed with six teeth; lacinia mobilis with seven teeth of differing sizes; palp broken off. *Lower lip* missing. *Maxilla 1* inner plate rounded and medium sized, reaching three quarters the length of outer plate, with two long slender plumose setae apically; outer plate topped with nine toothed robust setae; palp with one article, tipped with two long plumose setae and no facial slender setae. *Maxilla 2* missing. *Maxilliped* inner plate very short reaching only a quarter length of the outer plate, rounded, tipped with one robust seta and two slender plumose setae; outer plate not twisted around palp, reaching to two thirds along article 2 of palp, inner lateral margin lined with smooth robust setae becoming slender plumose setae distally, interspersed with small slender setae, palp broad, simple; article 2 broad, long bearing many strong slender plumose setae; article 3 ovoid, strongly setose, outer distal corner bearing two robust setae; article 4 slightly curved, acutely tipped with long robust seta, reaching two thirds the length of article 3.

***Pereon***: (Fig. 8) *Gnathopod 1* coxa reaching well past head anterior margin, coxa slightly expanded distally, ventral margin slightly produced forward, rounded, lined with long plumose slender setae, posteroventral corner with a small tooth, basis narrow, lateral margins lined with long plumose slender setae, medial setae long and plumose; merus weakly lobate and strongly setose particularly medially and posteriorly, carpus longer than merus (1.3×) and longer than propodus (1.2×), strongly setose, particularly medially and posteriorly with long plumose setae; propodus ovoid, subchelate, palm not well defined, angular, anterior and posterior margins and medially lined with long plumose slender setae, slight anterodistal lobe; dactylus short and curved, one third the length of propodus, inner margin lined with two slender setae and tipped with two robust setae. *Gnathopod 2* coxa similar length to coxa 1 (slightly shorter), ventral margin not angled, curved, fringed with many medial and marginal long plumose setae; basis long and narrow, posterior and anterior margin with fringe of long slender plumose setae, merus without lobe, long plumose setae on both anterior and posterior margins and medially; carpus considerably longer than merus (2.5×) and longer than propodus (1.8×), narrow and not lobate, both margins covered in long plumose setae, propodus narrow, ovoid, covered in long plumose setae, dactylus short, one third the length of propodus and slightly curved, inner margin smooth with four small plumose setae and tipped with one large robust seta.

***Pereopod 3*** coxa resembling coxa 2; basis long and narrow, anterior margin with short setal fringe, posterior margin without setae, merus narrow, shorter than basis (0.6×), longer than carpus and propodus together (1.4×), both margins with sparse long plumose setae; carpus with long plumose setae on posterior margin; propodus longer than carpus (2.0×), no setae on posterior margin, posterior margin slightly concave; dactylus long and narrow, slightly curved, slightly shorter than propodus (0.9×). *Pereopod 4* coxa trapezoid, posterior margin with extended rounded lobe, posterior margin below lobe straight, ventral margin evenly subquadrate and weakly setose, anteroventral corner rounded; basis shorter than coxa, posterior margin slightly serrate with fringe of long plumose setae along the whole length, anterior margin with short slender setae along length; ischium setose along posterior margin, merus long and narrow, shorter than basis (0.8×), longer than carpus and propodus together (1.5×), setose with plumose setae along complete length of posterior margin and distal half of anterior margin, carpus shorter than propodus (0.5×), setose along posterior margin; propodus long and narrow, setae on proximal posterior margin; dactylus long, narrow and slightly curved, shorter than (0.8×) propodus. *Pereopod 5* coxa with patch of long plumose setae on posterior corner; basis almost circular, anterior margin broadly rounded evenly, distally lined with long plumose setae, anterior margin slightly uneven, bi-sinusoidal, with reduced proximal distal lobe; ischium with acute posterior lobe; merus longer than ischium (2.0×), without setae on posterior margin, anterodistal corner with one robust seta, carpus longer than merus (1.5×), only setose along anterior margin (all plumose), slight posterodistal lobe, bearing two long strong, slender setae and one short robust seta; propodus and dactylus broken off. *Pereopod 6* basis nearly circular, anterior margin lined with short slender setae proximally and a few long plumose setae distally, posterior margin rounded, without setae, ischium with acute posterior lobe; merus longer than ischium (2.0×), not lobate, anterior margin fringed with six long slender setae and one robust seta distally, posterior margin without setae except one long slender seta distally; carpus longer than merus (1.4×) and longer than propodus (1.3×), anterior margin lined with eight robust setae, posterior margin without setae, distal corner produced distally with three long robust setae; propodus long and narrow, produced to form a very small distal lobe bearing two short slender setae, anterior margins with three short robust setae, dactylus very short, curved and smooth. *Pereopod 7* basis widest distally, posteroventral margin broadly rounded, lobe slightly exceeding the distal margin of the merus, medial surface densely setose, anterior margin with tiny slender setae, ventral and part of posterior margin lined with long plumose setae extending to junction with ischium, posterior corner smooth; ischium short and with two small setae on posterior margin, anterodistal corner bearing a robust seta; merus subequal to ischium, not lobate, anterior margin without setae, anterodistal corner bearing a robust seta, posterior with four long plumose setae and distally with three robust setae; carpus broken.

***Pleon***. (Figs 6, 9) *Epimeron 1* posteroventrally broadly rounded, ventral margin with plumose setae. *Epimeron 2* posteroventrally broadly rounded, ventral margin with plumose setae. *Epimeron 3* posteroventrally produced to form strong subacute tooth, posterior margin straight, ventral margin without setae.

***Urosome***: (Fig. 9) *Urosomite 1* strongly produced to form a distinct bilobed (in dorsal view) hood over urosomite 2. *Uropod 1* in situ reaching to tip of uropod 2 rami, peduncle longer than rami, outer margin lined with three short robust setae, inner margin lined with fine slender setae as a fringe and with three long plumose slender setae, distally with two robust setae, rami subequal in length, outer ramus lined with nine robust setae; inner ramus with four robust marginal setae. *Uropod 2* peduncle as long as rami, outer margin with four robust lateral setae, inner margin with three small and one long slender plumose setae, distally with three close together robust setae, inner ramus without setae and slightly longer than outer ramus, outer ramus with six short marginal robust setae, and one long subterminal plumose slender seta. *Uropod 3* peduncle shorter than rami, without robust setae, just one slender seta, ventral distal corner produced to form a tooth-like lobe; inner ramus slightly shorter than outer ramus, both rami narrow (broadest proximally); inner ramus lined with long slender plumose setae and one robust seta, outer ramus inner margin with long slender plumose setae, outer margin with five short slender setae laterally. *Telson* longer than wide (1.8×), 80% cleft; each lobe bilobed distally, with one slender apical seta, two robust laterally and two slender plumose setae laterally.

##### Variations.

Based on paratype, young female/male, 13 mm. SMF63345.

##### Urosome.

*Epimeron 3* posteroventral corner tooth more acute. Urosomite 1 more strongly bilobed. *Uropod 1* peduncular outer margin lined with three short robust setae, inner margin without setae, distally with two robust setae, outer ramus with two robust setae, inner ramus with three robust marginal setae. *Uropod 2* outer margin with four robust lateral setae, inner margin with two long and one small slender plumose setae, inner ramus without setae and slightly longer than outer ramus, outer ramus with two short marginal robust setae, and one long plumose slender seta. *Uropod 3* peduncle shorter than rami, with two narrow robust setae, ventral distal corner produced to form a tooth-like lobe; inner ramus slightly shorter than outer ramus, both rami narrow (broadest proximally); inner ramus with two long slender plumose setae and three robust setae, distally with one long and one short slender seta, outer ramus inner margin with two long slender, plumose setae, outer margin with five robust setae laterally. *Telson* longer than wide (1.8×), 80% cleft; each lobe bilobed distally, with one robust apical seta, one narrow, robust seta laterally.

##### Remarks.

Of the ten species of *Byblisoides* occurring in the world’s oceans, six are known from the Pacific Ocean. These species fall into the same traps as the rest of the ampeliscids, in that they have relatively similar morphology needing a combination of characters to provide differences. *Byblisoides jazdzewskae* sp. nov. has the closest affinities to *Byblisoides arcillis* and fits the most recent diagnosis of the genus (Peart 2018b). These similarities include the shape of the posterodistal lobe on the basis of pereopod 7 which is rounded, the shape of coxae 1 and 2, the bilobed nature of urosomite 1 and the shape of the telson. The two species differ by the shape of the anteroventral lobe of the head (rounded in the new species and subacute/acute in *B. arcillis*), the shape and strength of the bilobed nature of urosomite 1 (strongly bilobed and definitely rounded in the new species and acute and small in *B. arcillis*), and the length of the pereopod 7 posteroventral lobe which only extends just to the distal edge of the merus in the new species, while in *B. arcillis* it extends well into carpus. Unfortunately, the mouthparts of *Byblisoides arcillis* are not documented but the new species has the feature of one articulate maxilla 1 palp which does not appear in the other *Byblisoides* species that have the mouthparts documented.

##### Distribution.

Abyssal Pacific Ocean, Clarion-Clipperton Zone, 4111–4137 m.

##### Molecular data.

CO1 sequence data for the holotype of *Byblisoides jazdzewskae* sp. nov. is deposited in GenBank under accession number PQ734335. COI sequence data of the paratype is deposited in GenBank with the accession number PQ734288. The species has also received a Barcode Index Number from Barcode of Life Data Systems BOLD:AEB2414 (https://doi.org/10.5883/BOLD:AEB2414).

### Updated key to *Byblisoides* species

**Table:** 

1	Anterior edge of carpus of pereopod 7 with four long plumose setae	***B. juxtacornis* K.H. Barnard, 1931**
–	Anterior edge of carpus of pereopod 7 without plumose setae	**2**
2	Urosomite 1 not obviously produced to form a carina (from lateral view)	***B. esferis* J.L. Barnard, 1961**
–	Urosomite 1 obviously produced to form a carina (from lateral view)	**3**
3	Urosomite 1 carina strongly bilobed (from dorsal view)	**4**
–	Urosomite 1 carina not/ very weakly bilobed (from dorsal view)	**5**
4	Uropod 2 inner ramus with robust setae	***B. blasensis* J.L. Barnard, 1964**
–	Uropod 2 inner ramus without robust setae	**6**
5	Pereopod 7 basis posteroventral lobe extending halfway along carpus	***B. arcillis* J.L. Barnard, 1961**
–	Pereopod 7 basis posteroventral lobe only just extending to end of merus	***B. jazdzewskae* sp. nov**.
6	Pereopod 4 coxa ventral margin straight, anterior corner rounded	**7**
–	Pereopod 4 coxa ventral margin sinuous, anterior corner acute	***B. monicae* Peart, 2018**
7	Pereopod 7 basis ventral lobe acute	***B. plumicornis* Ledoyer, 1978**
–	Pereopod 7 basis ventral lobe rounded	**8**
8	Anteroventral corner of head produced	**9**
–	Anteroventral corner of head not produced	***B. profundi* Mills, 1971**
9	Anteroventral corner of head rounded	***B. bellansantiniae* Peart, 2018**
–	Anteroventral corner of head acute	***B. richardi* Peart, 2018**

## Supplementary Material

XML Treatment for Ampeliscidae

XML Treatment for Byblis

XML Treatment for Byblis hortonae

XML Treatment for Byblisoides

XML Treatment for Byblisoides jazdzewskae

## Footnotes

Species occurring in the Pacific Ocean.

## Additional information

### Author ORCIDs

Rachael A. Peart https://orcid.org/0000-0001-5968-0811

Eva C. D. Stewart https://orcid.org/0000-0001-8383-5705

### Data availability

All of the data that support the findings of this study are available in the main text.

## References

[B1] Barnard KH (1931) Diagnosis of new genera and species of amphipod Crustacea collected during the “Discovery” Investigations, 1925–1927. Annals and Magazine of Natural History 10(7): 425–430. 10.1080/00222933108673327

[B2] Barnard JL (1961) Gammaridean Amphipoda from depths of 400–6000 meters. Galathea Report 5: 23–128.

[B3] Barnard JL (1964) Deep-sea Amphipoda (Crustacea) collected by R.V. “Vema” in the eastern Pacific Ocean and the Caribbean and Mediterranean Seas. Bulletin of the American Museum of Natural History 127(1): 3–46.

[B4] Barnard JL (1966) Submarine canyons of southern California. Part V. Systematics: Amphipoda. Allan Hancock Pacific Expeditions 27(5): 1–166.

[B5] Barnard JL (1969) Gammaridean Amphipoda of the rocky intertidal of California: Monterey Bay to La Jolla. United States National Museum Bulletin 258: 1–230. 10.5479/si.03629236.258.1

[B6] Barnard JL, Karaman GS (1991) The families and genera of marine gammaridean Amphipoda (except marine gammaroids). Part 1. Records of the Australian Museum, Supplement 13(1): 1–417. 10.3853/j.0812-7387.13.1991.91

[B7] Bellan-Santini D, Dauvin JC (1993) Distribution and phylogeny of the genus *Byblis* Boeck (Ampeliscidae): preliminary statement. Journal of Natural History 27(4): 909–931. 10.1080/00222939300770561

[B8] Bellan-Santini D, Dauvin JC (2008) Contribution to knowledge of the genus *Haploops*, a new location for *Haploops lodo* (Crustacea: Amphipoda: Ampeliscidae) from the bathyal North Atlantic Ocean with a complement to the description of the species. Journal of Natural History 42(13–14): 1065–1077. 10.1080/00222930701877557

[B9] Boeck A (1871) Crustacea amphipoda borealia et arctica. Forhandlinger i Videnskabs-Selskabet i Christiania 1870: 83–280. 10.5962/bhl.title.2056

[B10] Dauvin JC, Sampaio L, Rodrigues AM, Quintino V (2021) Taxonomy and ecology of sympatric *Ampelisca* species (Crustacea, Amphipoda) from the Strait of Gibraltar to the Strait of Dover, North-Eastern Atlantic. Frontiers in Marine Science 8: 643078. 10.3389/fmars.2021.643078

[B11] Folmer O, Black M, Hoeh W, Lutz R, Vrijenhoek R (1994) DNA primers for amplification of mitochondrial cytochrome c oxidase subunit I from diverse metazoan invertebrates. Molecular Marine Biology and Biotechnology 35: 294–299. 7881515

[B12] Horton T, Lowry J, De Broyer C, Bellan-Santini D, Copilas-Ciocianu D, Corbari L, Costello MJ, Daneliya M, Dauvin J-C, Fišer C, Gasca R, Grabowski M, Guerra-García JM, Hendrycks E, Hughes L, Jaume D, Jazdzewski K, Kim Y-H, King R, Krapp-Schickel T, LeCroy S, Lörz A-N, Mamos T, Senna AR, Serejo C, Souza-Filho JF, Tandberg AH, Thomas JD, Thurston M, Vader W, Väinölä R, Valls Domedel G, Vonk R, White K, Zeidler W (2024) World Amphipoda Database. Ampeliscidae Krøyer, 1842. World Register of Marine Species. https://www.marinespecies.org/aphia.php?p=taxdetails&id=101364 [Accessed on 2025-04-02]

[B13] Jażdżewska AM, Biniek K, Martínez Arbizu P, Vink A (2025) Hidden behind the scene – high diversity, low connectivity of deep-sea Amphipoda in the polymetallic nodule fields in the central East Pacific. Biogeosciences, preprint. 10.5194/egusphere-2025-1794

[B14] Jażdżewska AM, Horton T (2026) New deep-sea Amphipoda from the Clarion-Clipperton Zone: 24 new species described under the Sustainable Seabed Knowledge Initiative: One Thousand Reasons campaign. In: Jażdżewska AM (Ed.) New deep-sea Amphipoda from Clarion-Clipperton Zone. ZooKeys 1274: 1–16. 10.3897/zookeys.1274.176711 PMC13040254 41929860

[B15] Kamanli SA, Kihara TC, Ball AD, Morritt D, Clark PF (2017) A 3D imaging and visualization workflow, using confocal microscopy and advanced image processing for brachyuran crab larvae. Journal of Microscopy 266(3): 307–323. doi: 10.1111/jmi.12540 28267872

[B16] Kearse M, Moir R, Wilson A, Stones-Havas S, Cheung M, Sturrock S, Buxton S, Cooper A, Markowitz S, Duran C, Thierer T, Ashton B, Meintjes P, Drummond A. (2012) Geneious Basic: An integrated and extendable desktop software platform for the organization and analysis of sequence data. Bioinformatics 28: 1647–1649. 10.1093/bioinformatics/bts199 PMC3371832 22543367

[B17] Krøyer H (1842) Nye nordiske slaegter og arter af amfipodernes orden, henhorende til familien Gammarina. (Forelobigt uddrag af et storre arbejde). Naturhistorisk Tidsskrift Ser. I. 4: 141–166.

[B18] Latreille PA (1816) Les Crustacés, les Arachnides, et les Insectes. In: [GLCFD] Cuvier. Le Règne Animal, Distribué d’après son Organisation, pour Servrir de Base a l’Histoire Naturelle des Animaux et d’Introduction a l’Anatomie Comparée. Volume 3: i-xxix+1–653. Paris: Deterville [Dated 1817, published 2 December 1816 fide Roux, 1976]

[B19] Ledoyer M (1976 [1978]) Contribution à l’étude des Amphipodes gammariens profonds de Madagascar (Crustacea). Téthys 8(4): 365–382.

[B20] Ledoyer M (1982) Crustacés Amphipodes Gammariens. Familles des Acanthonotozomatidae à Gammaridae. Faune de Madagascar 59(1): 1–598.

[B21] Liljeborg W (1856) Om Hafs-Crustaceer vid Kullaberg i Skane. Öfversigt af Kongl. Vetenskaps-Akademiens Förhhandlingar Stockholm 12(3): 117–138.

[B22] Lincoln RJ (1979) British marine Amphipoda: Gammaridea. British Museum (Natural History) 1–658.

[B23] Lörz AN, Tandberg AHS, Willassen E, Driskell A (2018) *Rhachotropis* (Eusiroidea, Amphipoda) from the North East Atlantic. ZooKeys 731: 75–101. 10.3897/zookeys.731.19814 PMC5810106 29472763

[B24] Lowry JK, Poore GCB (1985) The ampeliscid amphipods of south-eastern Australia (Crustacea). Records of the Australian Museum 36: 259–298. 10.3853/j.0067-1975.36.1985.348

[B25] Margulis RJ (1967) Deep-sea Ampeliscidae (Amphipoda, Gammaridea) from the Pacific Ocean. Crustaceana 13: 299–309. 10.1163/156854067X00486

[B26] Michels J, Büntzow M (2010) Assessment of Congo red as a fluorescence marker for the exoskeleton of small crustaceans and the cuticle of polychaetes. Journal of Microscopy (Oxford) 238(2): 95–101. 10.1111/j.1365-2818.2009.03360.x 20529057

[B27] Mills EL (1971) Deep-sea Amphipoda from the western north Atlantic Ocean. The family Ampeliscidae 1, 2. Limnology and Oceanography 16(2): 357–386. 10.4319/lo.1971.16.2.0357

[B28] Myers AA (2012) Amphipoda (Crustacea) from Palau, Micronesia: Families Ampeliscidae, Ampithoidae, Aoridae, Colomastigidae and Cyproideidae. ZooKeys 193: 1–25 10.3897/zookeys.193.3109 PMC3361137 22679377

[B29] Peart RA (2018a) Ampeliscidae (Crustacea, Amphipoda) from the IceAGE expeditions. ZooKeys 731: 145–173. 10.3897/zookeys.731.19948 PMC5799791 29416403

[B30] Peart RA (2018b) Antarctic and New Zealand *Byblisoides* (Amphipoda: Ampeliscidae) with a key to the world species. Zootaxa 4441(2): 347–365. 10.11646/zootaxa.4441.2.9 30314014

[B31] Poore GCB, Lowry JK (2023) New Australian species of Ampeliscidae (Crustacea: Amphipoda) from the Great Barrier Reef and eastern Australia with a key to Australian species. Records of the Australian Museum 75(4): 519–533. 10.3853/j.2201-4349.75.2023.1890

[B32] Ratnasingham S, Hebert PD (2007) BOLD: The Barcode of Life Data System (http://www.barcodinglife.org). Molecular Ecology Notes 7(3): 355–364. 10.1111/j.1471-8286.2007.01678.x PMC1890991 18784790

[B33] Ren X (1998) Studies on the family Ampeliscidae (Crustacea: Amphipoda) from Nansha Islands, South China Sea. Studies on Marine Fauna and Biogeography of the Nansha Islands and Neighbouring Waters. China Ocean Press, Beijing 3: 165–194.

[B34] Ren X (2006) Fauna Sinica, Invertebrata. Vol. 41, Crustacea, Amphipoda, Gammaridea (I). Science Press, Beijing, China, 588 pp.

[B35] Stebbing TRR (1906) Amphipoda. I. Gammaridea. Das Tiereich 21: 1–806. 10.5962/bhl.title.1224

